# Hyaluronan goes to great length

**DOI:** 10.15698/cst2020.09.231

**Published:** 2020-07-17

**Authors:** Vera Gorbunova, Masaki Takasugi, Andrei Seluanov

**Affiliations:** 1 University of Rochester, Departments of Biology and Medicine, Rochester, NY 14627, USA.

**Keywords:** hyaluronan, very high molecular weight hyaluronan, aging, longevity, naked mole rat

## Abstract

Hyaluronan is a major non-protein component of extracellular matrix that affects biomechanical properties of tissues and interacts with cell receptors. Hyaluronan is a linear glycosaminoglycan composed of repeating disaccharides of (β, 1–4)-glucuronic acid (GlcUA) and (β, 1-3)-N-acetyl glucosamine (GlcNAc). The length of hyaluronan can range from an oligomer to an extremely long form up to millions of daltons. The concept that emerged in the field is that high (HMW-HA) and low (LMW-HA) molecular weight hyaluronans have different biological properties and trigger different signaling cascades within the cells. LMW-HA is associated with inflammation, tissue injury and metastasis, while HMW-HA improves tissue homeostasis and has anti-inflammatory and antimetastatic properties. HMW-HA is used in the clinic to treat arthritis, and as a filler in surgery and in the form of rinses to treat local inflammation. However, HMW-HA products used in the clinic come in a range of sizes between 0.5-6 mDa that are used interchangeably. Remarkably, the tissues of a long-lived and cancer-resistant rodent, the naked mole rat, contain abundant HA of very high molecular weight. While human fibroblasts secrete HA up to 2 MDa, naked mole rat fibroblasts produce HA of 6-12 MDa. Does this very high HMW-HA (vHMW-HA) differ functionally from HMW-HA? We found that vHMW-HA has superior cytoprotective properties compared to HMW-HA, and interacts differently with the CD44 receptor leading to distinct transcriptional changes (Takasugi *et al.* (2020), Nat Commun). These results indicate that vHMW-HA has greater therapeutic benefits than the standard HMW-HA.

The naked mole rat is the longest-lived rodent with the maximum lifespan of over 35 years. Naked mole rats are protected from a wide range of age-related diseases including cancer, cardiovascular disease, arthritis and neurodegeneration. Remarkably, tissues of naked mole rats contain abundant vHMW-HA. We previously demonstrated that vHMW-HA inhibits proliferation of premalignant cells conferring cancer protection. It was unclear, however, whether vHMW-HA contributes to naked mole rat's resistance to other age-related diseases and ultimately to longevity.

HMW-HA was previously shown to protect cells from oxidative stress. The protective effect is dependent on hyaluronan receptor CD44. Takasugi *et al.* investigated whether there is a difference between HMW-HA and vHMW-HA in the ability to protect cells from oxidative stress. Importantly, we used physiological concentrations of hyaluronan, similar to the concentrations found in tissues (20 μg/ml). At this concentration HMW-HA, did not show a detectable cytopotective effect, while vHMW-HA protected human cells from cell cycle arrest and apoptosis induced by oxidative stress, doxorubicin, and γ-irradiation. The cytoprotective effect was dependent on CD44 receptor. Importantly, the cytoprotective effect disappeared when vHMW-HA was fragmented into shorter fragments, similar to HMW-HA.

Transcriptome analysis of human cells incubated with HMW-HA versus vHMW-HA revealed distinct differences. Genes differentially regulated included target genes of CD44-ICD, ELK1, and EGR1, which are downstream transcription factors of CD44. The most enriched transcription factor targets were targets of HIF1α, which overlap with CD44 targets. Many of the differentially regulated genes encoded regulators and interactors of p53. vHMW-HA attenuated activation of a subset of p53 target genes upon oxidative stress, which may explain the cytoprotective effect. Importantly, the cytoprotective effect of vHMW-HA was dependent on the presence of p53.

To understand how hyaluronan molecules that differ only by polymer length can trigger different pathways downstream of CD44 receptor we performed mass spectrometry analysis of CD44 interacting proteins in the presence of HMM-HA or vHMM-HA. We identified 99 proteins that were differentially associating with CD44 depending on hyaluronan length. For all of these proteins the interaction with CD44 was decreased in the presence of vHMW-HA. vHMW-HA also decreased association between CD44 molecules. These results suggest that vHMW-HA shields CD44 and reduces its interaction with other proteins, thereby altering the signaling pathways downstream of CD44 (**Figure 1**).

**Figure 1:**
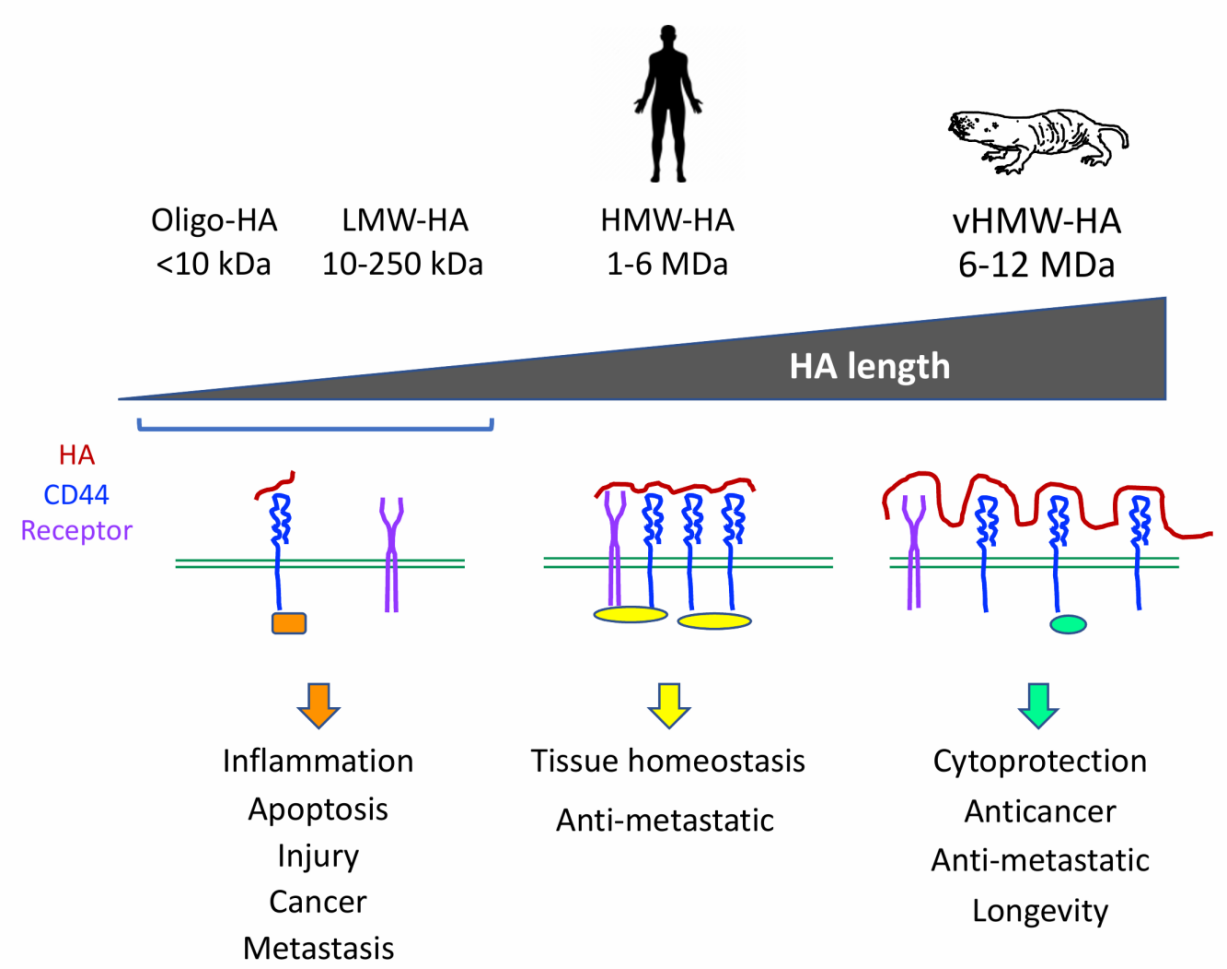
FIGURE 1: Biological effects of hyaluronans of different length. Hyaluronans of different length affect receptor interactions on cell surface and consequently engage different adaptor proteins (colored ovals and squares) on the cytoplasmic side activating distinct signaling pathways. Oligomeric hyaluronan (Oligo-HA) and low molecular weight hyaluronan (LMW-HA) do not cause clustering of CD44 (blue) and other receptors (purple). These hyaluronans are associated with inflammation, sites of injury, and promote cancer growth and metastasis. High molecular weight hyaluronan (HMW-HA) found in human tissues and in most other mammals, induces clustering of receptors and this signaling promotes tissue homeostasis, cell adhesion, and prevents metastasis by keeping cells in place. Very high molecular weight hyaluronan (vHMW-HA) is found in the naked mole rat. This hyaluronan shields CD44 receptor from interaction with other proteins leading to distinct downstream signaling characterized by cytoprotective effect, suppression of apoptosis. vHMW-HA has anticancer properties by limiting cell proliferation and metastasis and may ultimately promote longevity by protecting tissues from stress.

There are two major implications from these findings. First, our results suggest that vHMW-HA that is naturally produced by naked mole rat cells provides benefits beyond cancer protection. Since oxidative stress is believed to be one of the important contributing factors in aging process, the superior protective properties of vHMW-HA are likely to contribute to longevity and disease resistance of the naked mole rat. Apparently, longer is better for hyaluronan, and vHMW-HA produced by naked mole rat cells provides multiple benefits to these animals.

The second outcome has to do with the use of hyaluronan in medicine. Injectable hyaluronan is used widely as a treatment for osteoarthritis. Hyaluronan gels are used in eye surgery, and to treat burns and skin wounds. Eye drops containing hyaluronan are used to relieve dry eye symptoms; rinses containing hyaluronan are used to relieve pain and inflammation associated with ear, sinus and urinary tract infections. Finally, hyaluronan is used to treat aging skin, by injecting it into fascial wrinkles. These treatments use hyaluronan ranging from 0.5-6 MDa, and there is no consensus as to what size of hyaluronan is most beneficial. The choice of hyaluronan product is often determined by physician preference or insurance coverage, without scientific evidence. Our study strongly suggests that hyaluronan with sizes greater than 6 MDa has distinct biological benefits of protecting cells from stress. Clinically, this can translate to lower cell death, reduced inflammation, reduced swelling and other long-term benefits.

In summary, we argue that utilizing vHMW-HA in the clinic is likely to enhance the benefits of hyaluronan treatments. Since vHMW-HA is naturally only found in naked mole rats, it has not been used clinically, and commercially available hyaluronan products contain shorter hyaluronan. Exploring the clinical effects of vHMW-HA may bring the evolutionary adaptation that evolved in the long-lived and disease-resistant rodent to benefit human health.

